# A compendium and functional characterization of mammalian genes involved in adaptation to Arctic or Antarctic environments

**DOI:** 10.1186/s12863-017-0580-9

**Published:** 2017-12-28

**Authors:** Nikolay S. Yudin, Denis M. Larkin, Elena V. Ignatieva

**Affiliations:** 1 The Federal Research Center Institute of Cytology and Genetics, The Siberian Branch of the Russian Academy of Sciences, 630090 Novosibirsk, Russia; 2 Novosibirsk State University, 630090 Novosibirsk, Russia; 3 The Royal Veterinary College, University of London, London, NW1 0TU UK

**Keywords:** Cold, Adaptation, Mammal, Database, Genome, Gene, Positive selection

## Abstract

**Background:**

Many mammals are well adapted to surviving in extremely cold environments. These species have likely accumulated genetic changes that help them efficiently cope with low temperatures. It is not known whether the same genes related to cold adaptation in one species would be under selection in another species. The aims of this study therefore were: to create a compendium of mammalian genes related to adaptations to a low temperature environment; to identify genes related to cold tolerance that have been subjected to independent positive selection in several species; to determine promising candidate genes/pathways/organs for further empirical research on cold adaptation in mammals.

**Results:**

After a search for publications containing keywords: “whole genome”, “transcriptome or exome sequencing data”, and “genome-wide genotyping array data” authors looked for information related to genetic signatures ascribable to positive selection in Arctic or Antarctic mammalian species. Publications related to Human, Arctic fox, Yakut horse, Mammoth, Polar bear, and Minke whale were chosen. The compendium of genes that potentially underwent positive selection in >1 of these six species consisted of 416 genes. Twelve of them showed traces of positive selection in three species. Gene ontology term enrichment analysis of 416 genes from the compendium has revealed 13 terms relevant to the scope of this study. We found that enriched terms were relevant to three major groups: terms associated with collagen proteins and the extracellular matrix; terms associated with the anatomy and physiology of cilium; terms associated with docking. We further revealed that genes from compendium were over-represented in the lists of genes expressed in the lung and liver.

**Conclusions:**

A compendium combining mammalian genes involved in adaptation to cold environment was designed, based on the intersection of positively selected genes from six Arctic and Antarctic species. The compendium contained 416 genes that have been positively selected in at least two species. However, we did not reveal any positively selected genes that would be related to cold adaptation in all species from our list. But, our work points to several strong candidate genes involved in mechanisms and biochemical pathways related to cold adaptation response in different species.

**Electronic supplementary material:**

The online version of this article (10.1186/s12863-017-0580-9) contains supplementary material, which is available to authorized users.

## Background

Millions of years of natural selection in the Arctic and Antarctic environments have made a few mammalian species well adapted to survive in these extreme cold environments [1, 2]. It is expected that the species adapted to surviving in Arctic and Antarctic conditions have accumulated genetic changes that help them to cope with the cold.

Homeothermic (able to keep their body temperature at the same level despite changes in the environmental temperature) mammals use three basic strategies to cope with low temperatures: (1) increased heat production via shivering and non-shivering thermogenesis; (2) reduced heat loss from the body surface with the help of fur and/or subcutaneous adipose tissue; (3) hibernation to temporarily achieve and survive low body temperature [3]. While physiological responses to low temperatures are well studied [4, 5], the exact molecular mechanisms behind them are still unknown. In the era of genomics, an important step is to reveal the genomic regions under positive selection that control physiological performance. This needs to be done to identify genes and gene pathways controlling organism responses to environmental temperature changes.

There are some studies concerning the aspects of cold adaptation and genes possibly involved in these processes. Sazzini et al. [6] investigated signatures of cold adaptation within 28 genes involved in this functional pathway in modern and archaic humans. They found that patterns of variations within the leptin receptor (*LEPR*) gene involved in the increased heat dissipation by mitochondria have been affected by positive selection in modern East Asians, but not in Europeans. Hancock et al. [7] used an evolutionary approach to test the hypothesis that variants associated with the increased expression of *uncoupling protein UCP)* genes are related to adaptation to severe winter climates. Uncoupling protein 1 (UCP1), a mitochondrial protein expressed in brown adipocytes in mammals, plays a central role in non-shivering thermogenesis allowing the dissipation as heat of the proton gradient generated by the respiratory chain and thereby uncoupling respiration and oxidative phosphorylation (ATP production). Authors indeed found a significant correlation of *UCP1* substitutions -3826G/A and *UCP3*-55C/T with severe winter climate residence in a panel of 52 worldwide populations. Quagliariello et al. [8] used the genetic information for 21 genes involved in nutritional and thermoregulation processes among three Western European populations and suggested that multiple selection events at the *PRDM16* functional pathway shaped the adaptation of western Europeans to different climate conditions. The transcriptional regulator, PR domain zinc finger protein 16 (PRDM16), determines the development of brown adipocytes from a progenitor. Thus, modulation of non-shivering thermogenesis, for which the most specialized cells are the brown adipose tissue adipocytes might have driven the cold climate adaptation in human populations.

Wollenberg et al. [9] did a chicken multimodal network related to thermal adaptation built on functional relationships of markers from the human, chicken, and lizard genomes. To investigate if thermal adaptation gene candidates are functionally closer related to each other than similar genes that were not proposed as adaptation candidates, authors calculated and compared clustering coefficients, heterogeneity, network density etc. The network created by Wollenberg et al. was better organized into functional pathways, functionally associated, and faster in information exchange than it would be expected by random chance.

Identification of loci under positive selection often leads to the detection of dozens of candidate genes [10–12]. Functional significance of only a small number of genetic variants from these genes can be verified experimentally, due to the high cost of verification and the very small phenotypic effects of individual variants. Functional significance testing implies a quantitative comparison of a level of adaptation and/or other related characteristics (e.g. gene expression levels) in individuals bearing different (or combinations of) genetic variants in vivo [13, 14]. Of course, functional testing in a natural environment would be optimal, but not feasible for most species due to financial, logistical, legal, and ethical complications. Such an analysis can be performed using directed genome editing with the CRISP-Cas9 system [15], but this also requires considerable effort. Therefore, for further experimental verification, it is necessary to select “key” genes with a preferably large effect, such as genes found shared in several independent studies within a species or in multiple species [16].

There are some studies of Arctic adaptation, which have compared genomic divergence between two closely related species adapted to different environmental conditions to identify the candidate genes that are relevant to cold adaptation [17–20]. It is still unclear whether the same genes might be involved in cold tolerance in different species and if there are common genetic mechanisms of cold adaptation.

The aim of this work, therefore, was to create a compendium of genes that underwent positive selection as a result of mammalian adaptations to cold environments, to identify common genes related to cold tolerance and mechanisms of cold adaptation shared by several mammals, and to determine promising candidate genes, biological processes, and organs for further in vitro studies.

## Methods

### Revealing overlapping sets of genes from published datasets originating from different species

Searches in the PubMed database were made on 03/10/2017 with the following requests: "Cold adaptation AND Mammal AND Genome", “Cold AND Genomics”, “Cold AND Transcriptomics”, “Cold AND Bioinformatics”. “Low temperature” was also used instead of “cold”. Further, papers published no earlier than the year 2010 were used (after the widespread use of next-generation sequencing technologies). The selection criteria had to include publications that involved Arctic or Antarctic species of mammals and: a) contained a whole genome, transcriptome, exome sequencing data, or genome-wide genotyping array data; or b) included the results of a search for signatures of positive natural selection.

From each publication, lists of genes potentially involved in adaptation to cold climate were extracted. In order to build intersections between lists of genes from different species, we converted all gene names to the human official gene symbols when performing queries to the NCBI’s Entrez Gene database (www.ncbi.nlm.nih.gov/gene). To calculate the number of intersections between lists of genes we used a web tool (http://bioinformatics.psb.ugent.be/webtools/Venn/) developed by the Bioinformatics and Evolutionary Genomics group (Vlaams Instituut voor Biotechnologie/Ghent University, Belgium).

### Gene ontology enrichment analysis

Gene Ontology (GO) term enrichment analysis enables functional characterization of gene sets revealed using various criteria. It is widely used in studies aimed at dissecting the genetic bases of complex phenotypic traits (e.g. physiological, pathological or adaptive) [20–22]. The benefits of GO term enrichment analysis for functional characterization of gene sets have been demonstrated in our previous publications [23–26].

Biological functions of the 416 genes that likely underwent positive natural selection in at least two species and two sets of tissue-specific genes revealed by the TSEA (Tissue Specific Expression Analysis) tool [27] were revealed using the GOrilla online enrichment tool (Gene Ontology enRIchment anaLysis and visuaLizAtion Tool; [28]). Based on a complete theoretical characterization of the underlying distribution, GOrilla tool provides a list of enriched GO terms in a target set versus a background set using a hypergeometric model. We used a list of 28,149 human genes as a background set. The GO enrichments were performed independently for the following categories: Biological Processes, Molecular Functions, and Cellular Components. To control for multiple testing errors *p*-value and FDR q-value were applied. If the reported raw *p*-value was <0.001 the corresponding GO term was considered to be enriched [29].

### Gene expression analysis

The GOrilla tool enables to organize and condense large gene lists into biologically meaningful modules, but it relies on manually curated information from the GO database, which might be not unbiased. Some hub genes are associated with multiple GO terms while a substantial portion of genes are not annotated at all. For example, according to GOrilla, only 18,433 of all human genes are associated with at least one GO term. We assumed, therefore, that GOrilla tool outcomes may not be free from both type 1 and type 2 errors. To obtain an additional independent functional characterization of genes from our compendium, we used another approach, which is based on a dynamic source of information, such as gene expression across tissues.

We used the TSEA tool in order to perform gene expression analysis based on human tissue expression data. This tool utilizes the data collected as part of the Genotype-Tissue Expression project. This project included RNA-seq data from 45 tissues collected from 189 human individuals. The TSEA tool identifies the overlap between a user-supplied gene list and a reference gene list applying different tissue specificity thresholds and finally estimates statistical significance of the overlap using the Fisher’s exact test with the Benjamini-Hochberg correction [27].

## Results

### The compendium of genes involved in the adaptation to a cold environment

Publications on signatures of positive selection in six species of Arctic and Antarctic mammals were selected, namely Human, Arctic fox, Yakut horse, Mammoth, Polar bear and Minke whale. Lists of genes with signatures of positive selection extracted from these publications are provided in the Appendix (Additional file 1: Tables S1-S7).

The human gene set was compiled based on two publications, due to the terms that had been used in the PubMed search being restrictive. In the first study, the analysis of exome sequencing data from 18 Greenlandic Inuit individuals and an Illumina MetaboChip array genotyping data from 191 Inuit individuals was used to compute the population branch statistic (PBS) test and revealed signatures of positive selection in 16 genes [30]. In the second study, Cardona et al. [31] has conducted single nucleotide polymorphism (SNP) genotyping of representatives of ten indigenous Siberian populations for 730,525 SNPs and searched for signatures of positive selection using the integrated Haplotype Score (iHS), Cross-Population Extended Haplotype Homozygosity (XP-EHH) and PBS tests [31]. A list of 46 genes related to cold climate adaptation was obtained by combining the top 1% iHS, XP-EHH and PBS-positive genomic regions putatively targeted by selection in the genomes of Siberian populations with the data reported by Hancock et al. [32], which revealed SNP loci with a strong correlation between the allele frequencies and minimum winter temperatures in 61 worldwide human populations. For further analysis, we used a combined list (59 genes), obtained after merging Fumangalli’s (16 genes) and Cardona’s (46 genes) lists (the number of common genes were three) (Additional file 1: Table S1).

Arctic fox gene set was extracted from the report presenting the analysis of three transcriptome libraries generated from pooled RNA-extracts of three different tissues (liver, brain, and kidney) from two Arctic fox individuals and one Red fox. A total of 35 genes affected by positive selection in the Arctic fox were identified by the branch site test (Additional file 1: Table S2) [18].

Yakut horse gene set was based on Libardo et al. [19], reporting analysis of complete genomes of nine modern and two ancient Yakut horses compared with the genomes of a modern horse and late Pleistocene horse. It was shown that the native horse that inhabited the Yakut region in the Holocene was not an ancestor of the modern Yakut horse, but possibly its ancestor was introduced to the region by migrating Yakut people several centuries ago [19]. Thus, Yakut horse can be considered as an example of the most rapid adaptation to the extremely cold Arctic climate. Traces of positive selection were revealed using the F_ST_-outlier approach, which allowed the authors to identify 134 genes with signatures of selection (Additional file 1: Table S3).

Mammoth gene set resulted from a comparison of the whole genome sequences of two Mammoths and three Asian elephants. This has shown that mammoths differ from elephants in fixed amino acid substitutions found in 3207 genes (Additional file 1: Table S4) [20].

Polar bear gene set consisted of the data originating from 89 sequenced genomes of Polar and Brown bears [17]. Traces of positive natural selection in the Polar bear lineage were revealed using four statistical tests. Since the authors did not specify the lower value of the confidence threshold, in our work we used 1100 genes (Additional file 1: Table S5) with a *p*-value threshold of 0.05 as defined by the Hudson-Aguade-Kreitman (HKA) test.

For the Minke whale gene set we used 278 genes that were found to be subjected to positive selection (Additional file 1: Table S6) in four Minke whale genomes using branch-site likelihood ratio tests for single-copy gene families with a 10% FDR cutoff [33]. The Minke whale was included into our study, because it is believed that it has been adapted well to life in cold water [34].

**Fig. 1:**
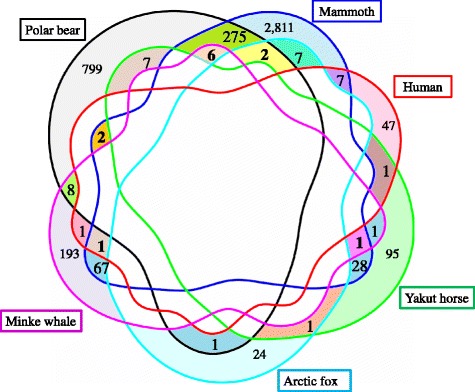
Six-way Venn diagram depicting overlaps between genes with signatures of positive selection from genomes of six Arctic mammals. Twelve genes that had traces of selection in three species (found at the intersections of any three gene sets) are marked with bold numbers

For further analysis, all gene names obtained from publications were unified in accordance with the official gene symbols of human orthologs (Additional file 1: Tables S1-S6). In total, we obtained 4394 unique genes from seven research articles (data not shown, *Selection_all* list). The list of 416 genes that potentially underwent positive natural selection in at least two different species (Fig. 1, Additional file 1: Table S7) is referred below as the *Selection_shared* list. Twelve of these 416 genes (*DSP, SLC38A4, TCOF1, SFI1, EXPH5, ICAM4, KNG1, NEB, ZDBF2, SELPLG, FAM208B,* and *SPTBN5*) had traces of positive selection in three species. There were no genes that had traces of natural selection in more than three species.

It is worth mentioning that there was no correlation between the overlap of genes and species phylogeny. Of the six species we studied, five were from different Orders of mammals. Only the Arctic fox and the Polar bear were from different families of the same Order – the Carnivora. However, a notable portion of genes under positive selection in the Polar bear overlapped with genes of Mammoth (285 out of 4307), while only three genes out of the total of 1135 overlapped between the Polar bear and Arctic fox (chi-square test *p* < 0.00001).

### Gene ontology term enrichment analysis of genes from the compendium

To test the hypothesis that the compendium comprising of 416 genes found at the intersections of at least two gene sets was enriched in some functional groups of genes we performed GO analysis using the GOrilla tool. We found enrichment in 13 GO functional classes (Additional file 1: Table S8). Fold enrichments exceeded 1.5 for all these GO classes and *p*-values were ≤0.001. Many of these GO classes were from the top-level hierarchy (“multicellular organismal catabolic process”, “multicellular organism metabolic process”, “multicellular organismal macromolecule metabolic process”, “extracellular structure organization”, and “regulation of microtubule-based movement”) and, therefore, highlighted general processes, and for this reason will not be discussed in detail.

The revealed GO terms relevant to the scope of the present study may be divided into three main groups: group I) includes terms associated with collagen proteins and extracellular matrix, namely “collagen catabolic process”, “extracellular matrix organization” etc.; group II) includes terms associated with anatomy and physiology of cilium: “regulation of cilium beat frequency”, “regulation of cilium movement”, “non-motile cilium assembly”; group III) includes terms associated with docking: “ciliary basal body docking” and “membrane docking”.

### Gene expression analysis

The next step was to identify tissue-specific genes, corresponding tissues and organs using the TSEA tool. All 416 genes from the *Selection_shared* list were analyzed. We found that genes from the *Selection_shared* list were over-represented in tissue-enriched TSEA lists of genes expressed in the lung (pSI threshold <0.05) and liver (pSI threshold <0.01). According to TSEA, 42 genes were found at the intersection between the *Selection_shared* list and the list of genes enriched in the lung at a pSI threshold of <0.05. In addition, 21 genes were found at the intersection between the *Selection_shared* list and the list of genes enriched in the liver at a pSI threshold of <0.01 (Additional file 1: Table S9).

To reveal functional characteristics of these two subsets of tissue-enriched genes comprising 42 and 21 genes respectively, we performed a GO enrichment analysis using the GOrilla tool, though numbers are probably too low to enable robust enrichment analysis. The results of GO analysis are presented in Additional file 1: Tables S10 and S11, respectively. GOrilla revealed 11 and 24 over-represented GO terms characterizing these two sets of genes from the compendium, which were expressed in a tissue-enriched manner in the lung and liver, respectively.

## Discussion

### The compendium of genes controlling adaptation to cold environments

To obtain a systematic overview of genes contributing to adaptation to cold environments, which may serve as targets for further experimental verification, we created a compendium of genes showing signatures of positive selection from genomes of six mammals dwelling in/near the Arctic or Antarctica: Human, Arctic fox, Yakut horse, Mammoth, Polar bear, and Minke whale. At present, the compendium contains information on 416 genes that potentially underwent positive natural selection in at least two mammalian species (Additional file 1: Table S7).

As we were interested in finding genes under selection in populations surviving in extreme cold conditions, our selection of species/populations was somewhat restrictive. This is especially relevant to the human populations. The presented gene list likely misses candidate loci detected in studies focused on human populations dwelling in less extreme environments than the Arctic. In fact, other candidate genes have been recently identified as putative targets of positive or balancing selection ascribable to cold-related selective pressures [6, 8, 35], even through the examination of human populations living in less extreme environments than the Siberian population. In fact, the mentioned studies provided evidence for mechanisms plausibly evolved to cope with cold environments in some East Asian and European populations, which differ from those evolved by Siberian groups. Moreover, humans, unlike other animals, are to some extent able to avoid the damaging effect of cold: they build dwellings, sew clothes, etc. Interestingly, among the humans it is the aborigines of Australia, not the Northern populations, who are considered to be the most adapted to cold: the energy cost of their physiological responses to moderate cold are lower than in the Europeans and Eskimo [36, 37].

Previously, another compendium has been created comprising of 113 shared low temperature response proteins/gene products, each of which was found in at least two eukaryotic species [38]. This compendium was built based on 2030 low temperature response protein/gene product entries, of which 1353 were up-regulated and 549 were down-regulated in response to various cold exposures across 34 different eukaryotic species (e.g. plants or mammals). Of these shared proteins/gene products, only 58 were independently either up or down regulated across species. The authors considered only acute cold effects. According to Carrasco et al. [38] there are two different responses to low temperature: (1) acclimation, the process in which an individual organism adjusts to a cold environment within its lifetime; and (2) adaptation as a result of cold acting as a selective pressure over many generations. A list of candidate genes for cold acclimation may be quite different from a list for cold adaptation.

Another group has tested the hypothesis that previously identified candidate genes for thermal adaptation are functionally related via gene interaction pathways [9]. To test this hypothesis, Wollenberg et al. [9] has integrated literature searches of known markers for thermal adaptation with functional relation modelling. The aim of this study was to identify genes that were found to be under natural selection or showed a short-term response to changes in the thermal environment. Surprisingly, 44 candidate markers initially identified from diverse lineages of vertebrates such as Human and fish were all in closer functional relationships with each other than it would be expected by random chance. According to the authors’ view, this suggested that the general genetic functional network for thermoregulation and/or thermal adaptation to the environment might be regulated via similar evolutionarily conserved pathways in different vertebrate lineages.

In the current study, we have identified 12 genes that potentially underwent positive natural selection in three species of mammals. These 12 genes were found at the intersections of Human, Polar bear, and Mammoth lists (2 genes), Polar bear, Mammoth, and Arctic fox lists (2 genes), Polar bear, Mammoth and Minke whale lists (6 genes), Human, Mammoth, and Minke whale lists (1 gene), and Yakut horse, Mammoth, and Minke whale lists (1 gene) (Fig. 1). We found that four out of these 12 genes (*SPTBN5, KNG1, ICAM4,* and *DSP*) were directly related to cardiovascular function.

The *SPTBN5* gene encodes a spectrin protein that plays an important role in the maintenance of plasma membrane integrity and cytoskeletal structure stability. Spectrin tetramers bind to actin microfilaments and form nets lining the inner surface of the plasma membrane in many cell types [39]. SNPs in the *SPTBN5* gene have been associated with stroke incidence in a Japanese population, but only in patients with metabolic syndromes [40].

The *KNG1* kininogen gene encodes two polypeptides, high molecular weight and low molecular weight kininogens, which are formed by alternative splicing and represent potent cysteine protease inhibitors. High molecular weight kininogen takes part in the activation of the factor XII of blood coagulation system [41]. High molecular weight kininogen also is a precursor of the bradykinin peptide. Bradykinin modulates cold-sensing TRPM8 channel by inhibiting the channel and shifting its activation threshold to colder temperatures [42]. It has been reported that stimulation of this channel mediates brown adipose tissue thermogenesis [43].

The *ICAM4* gene encodes the Landsteiner-Wiener blood group antigen(s) that belong to the immunoglobulin superfamily, and that shares similarity with the intercellular adhesion molecule (ICAM) protein family. ICAM4 is likely to contribute to red cell adhesion in a variety of settings, including hematopoiesis, as well as vascular disorders [44]. The best documentation of a pathophysiological role for ICAM4 in human disease is in sickle cell disease, where it contributes to red cell adhesion to endothelial cells and the development of vaso-occlusion, the hallmark of that disease. ICAM4 may also contribute to other intravascular processes, such as both venous and arterial thrombosis, due to its ability to interact with both activated platelets and leukocytes.

The desmoplakin gene (*DSP*) encodes a basic protein of desmosomes, the intercellular junctions that mechanically attach adjacent epithelial cells and cardiac muscle cells. Intermediate microfilaments of the cell are attached to the desmosome via membrane protein complexes assembled from proteins encoded by the *DSP* gene [45]. Researchers have associated mutations in the *DSP* gene with severe cardiomyopathy [46] and sudden night-time death [47], as well as dermatitis and palmoplantar keratoderma [48]. The latter disease is associated with thinning of the skin on the palms and soles. Therefore, the role of *DSP* gene in cold adaptation can be associated with both the need to increase the thickness and thermal insulation of the skin layer on the limbs, and with an increased functional load on the cardiovascular system.

Analogous to the *DSP* gene, natural selection of *EXPH5* gene variants may have been associated with improvement of mechanical properties of the skin: the Exophilin 5 protein, encoded by the *EXPH5* gene, is involved in the secretion of exosomes into the extracellular space [49]. Reduced expression of Exophilin 5 protein results in keratin filament defects of the skin [50]. Mutations in *EXPH5* are associated with a disorder called epidermolysis bullosa, leading to skin fragility [51].

The *SLC38A4* and *SFI1* genes are involved in cold adaptation, supposedly, due to their impact on basal metabolism. The *SLC38A4* gene encodes a transporter protein that is expressed predominantly in the liver and participates in the transfer of both cations and neutral amino acids [52]. It is believed that this protein plays a significant role in gluconeogenesis, since SNPs in the *SLC38A4* gene are found to be associated with hyperglycemia [53]. The complex of the *SFI1* gene product and the centrin protein (CETN2) forms contractile filaments that attach to centrosomes during cell division [54]. In humans, an association of SNPs in the *SFI1* gene with levels of glycosylated hemoglobin (a marker for average blood glucose level) has been demonstrated [55].

It is well known that airway congestion plays an important role in protection against heat loss during cold adaptation [56]. The *TCOF1* gene encodes a nucleolar protein, which is involved in ribosomal DNA gene transcription through its interaction with upstream binding transcription factor (UBF). Mutations in this gene have been associated with Treacher Collins syndrome, a disorder which includes abnormal craniofacial development [57]. This condition is characterized among others by airway problems, secondary to mandibular and pharyngeal hypoplasia, small or obstructed nasal passages etc.

The *SELPLG* gene (or *PSGL-1*) encodes a glycoprotein, which is a ligand for the cell adhesion molecules (P-, E- and L-selectin) expressed in myeloid cells and activated T lymphocytes. SELPLG protein plays a crucial role in the migration of leukocytes to sites of inflammation [58]. *SELPLG* knock-out mice are susceptible to infection by streptococcus [59]. The role of the *SELPLG* gene in cold adaptation can be associated with resistance to pneumonia and other infections.

The role of the *NEB* gene in cold adaptation appears to be related to its contribution to heat production via shivering thermogenesis. The *NEB* gene encodes nebulin protein, which in complex with actin, forms fine filaments in skeletal muscle sarcomeres. It is believed that nebulin regulates actin-myosin interaction through inhibition of ATPase activity with involvement of calcium ions [60]. Mutations in the *NEB* gene have been identified as a cause of nemaline myopathy, an autosomal recessive disorder [61]. Mice deficient in the nebulin protein have shortened fine filaments and abnormal contractile properties of muscles [62].

The *ZDBF2* gene encodes a protein containing DBF4-type zinc finger domains, so potentially it may bind DNA, RNA, protein and/or lipid substrates. Human *ZDBF2* gene was mapped to chromosome 2q33.3 and was paternally expressed in lymphocytes but bi-allelically expressed in the placenta [63]. Previous studies reported that maternal and paternal uniparental isodisomies for human chromosome 2 were responsible for various genetic abnormalities [64]. It is possible that positive selection in the *ZDBF2* gene is associated with some yet known physiological or immunological adaptations of animals to low temperatures.

Currently, the function of the *FAM208B* gene is unknown. Some genes from its FAM family were associated with osteosarcoma in humans [65] and some were a part of the Autism Spliceform Interaction Network [66].

### Gene ontology enrichment analysis of genes from the compendium

In this study, the GOrilla enrichment tool was used for gene ontology enrichment analysis, using two lists of genes as input (target *Selection_shared* list and background list of all human genes). Thirteen significantly enriched functional categories (*p*-value of enrichment ranging from 3.77 * 10^−6^ to 9.68 * 10^−4^) were selected for gaining insights into biological mechanisms of adaptation to cold environments in mammals. FDR corrected *p*-values for multiple testing using the Benjamini and Hochberg method (FDR q-value) were found insignificant in all classes, so the results of our analysis should be considered as explorative.

The class “Multicellular organismal catabolic process” included *COL7A1, COL5A3, COL4A4, COL10A1, COL6A6, COL6A1* collagen genes as well as extracellular matrix-remodeling genes *MMP1, ADAMTS14*, and *MMP27*. Prominent suppression of collagen gene expression was observed during cold-induced remodeling (increase in relative heart mass and increase in ventricular myocyte size) in the heart of rainbow trout [67]. Cold-acclimated zebrafish had increased expression of the gene transcript for matrix metalloproteinases in the connective tissue of the heart [68]. The proportion of collagen in the body was lower in mice kept at −3 °C for up to twenty-nine generations [69]. Increased matrix metalloproteinase-2 (MMP2) expression, activation and activity was observed in the heart of broiler chickens reared in cold conditions [70].

Some other noteworthy classes were “collagen catabolic process”, “collagen metabolic process”, “multicellular organism metabolic process”, “multicellular organismal macromolecule metabolic process”, “extracellular matrix organization”, and “extracellular structure organization, extracellular matrix”. They also included genes *COL7A1, MMP1, ADAMTS14*, and *COL6A1*, that play important roles in remodeling and maintaining extracellular matrix integrity (see above).

A number of GO terms were associated with the functions of cilia – “regulation of cilium beat frequency”, “regulation of cilium movement”, and “ciliary basal body docking”. Probably the frequency of beating of the cilia has an adaptive value in the cold, because some authors connected the beating of cilia with respiratory function [71, 72].

### Gene expression analysis

Gene expression analysis with the TSEA tool has revealed significant intersections (Benjamini-Hochberg corrected *p*-value <0.05) between the *Selection_shared* list and the TSEA lists of tissue-enriched transcripts from the lung and liver. This observation suggests that liver and lung may play an important role in adaptations to cold environments. Identification of the most represented GO terms characterizing tissue-enriched genes from the lung and liver has provided further clues into the important role of the immune system in cold adaptation. The prominent functional terms have included: (1) “Regulation of phagocytosis”, “Complement activation, classical pathway”, “Acute-phase response” (liver), (2) “Defense response”, “Immune response”, and “Respiratory burst” (lung). We identified one gene (*C4BPA*, encoding complement component 4 binding protein) that participates in all of the above biological processes, which indicates the important role of the complement system. Because both the gene lists analyzed at this step contained small numbers of genes (42 genes for lung and 21 genes for liver), the related findings need to considered with caution. Nevertheless, because of a high relevance of our results to previous findings [56, 73], we propose that all enriched GO terms should be kept in mind as relevant to adaptations to cold environments.

Arctic (Antarctic) air is known to be cold and dry. When inhaled cold dry air leads to cooling of the airways and hyperosmolarity of the fluid for the lining of the respiratory tract in the lungs. The patterns of respiration are different in cold-adapted organisms and in organisms not adapted to cold, in thermoneutral conditions and under the cold exposure. Iakimenko et al. [74] compared the parameters of the human breathing in two experimental groups (adapted and not adapted to cold) in different conditions (thermoneutral and testing cooling) and found difference between the acute cold effect and adaptation to cold. In healthy young males from Western Siberia during winter pulmonary ventilation becomes limited and the number of functioning lung units is reduced. At the same time, for providing compensation, lung diffusion capacity increases [75]. In livestock animals, a decrease in ambient temperature elicits a change in breathing pattern, favoring increased tidal volume and decreased deadspace ventilation, thus minimizing respiratory heat loss [76–78]. Pulmonary mechanics are worsened due to bronchoconstriction, airway congestion, secretions and decreased mucociliary clearance [56]. These processes are possibly responsible for decreased immune function and protection against airborne pollutants.

In accordance with the above-mentioned processes, we found enrichment in six GO functional terms relevant mainly to immune function (“defense response”, “immune response”, “respiratory burst”, “immune system process”, “receptor activity”, and “molecular transducer activity”). They included genes responsible for the cellular (*CD163*, *PKHD1L1*) and humoral (*CD180*) immunity, complement system (*C4BPA*, *C5AR1*), cytokine signaling (*IL1A*) etc. Exposure to cold has often been associated with increased incidence and severity of respiratory tract infections in human [79].

Another three classes characterized the mucociliary clearance in lungs (“regulation of cilium beat frequency”, “regulation of cilium movement”, and “regulation of microtubule-based movement”). All these terms included only the *CATSPER1* and *DNAAF1* genes. The protein encoded by the *DNAAF1* gene is cilium-specific and is required for the stability of the ciliary architecture. It is involved in the regulation of microtubule-based cilia and actin-based brush border microvilli. Mutations in this gene are associated with primary ciliary dyskinesia [80]. The CATSPER1 protein belongs to a family of putative cation channels that are specific mainly to spermatozoa and localize to the flagellum [81]. The enrichment of the list of genes expressed in the lung with these terms may be also associated with the change in surfactant properties during adaptation to low temperatures [82].

There is strong evidence that liver has an important role during cold acclimation/adaptation. Cold exposure in rats was shown to increase liver temperature, total liver and mitochondrial mass, respiration capacity of hepatocytes, and hepatic gluconeogenesis [83–85]. The energy state of rat liver (ATP level, adenine nucleotide pool, phosphorylation potential) differs significantly at various times of cold acclimation [86]. Cold adaptation at the liver level could provide brown adipose tissue with glucose and fatty acids from very low density lipoproteins [83]. Recently Simcox et al. [87] identified acylcarnitines as a novel source of energy for brown fat thermogenesis in mice and showed that in response to cold, the liver activates a transcriptional program through the nuclear receptor HNF4a to increase acylcarnitine production. Blocking hepatic acylcarnitine synthesis impairs adaptive thermogenesis.

The GO enrichment analysis of the TSEA list of genes expressed in the liver revealed two terms related to immunity - “regulation of phagocytosis” and “complement activation” (*C4BPA, MBL2, AHSG,* and *C6* genes). Polarized M1 (pro-inflammatory) macrophages are known to cause adipose tissue inflammation, whereas polarized M2 (anti-inflammatory) macrophages promote white adipose tissue remodelling into brown adipose tissue (so called “browning/beiging”), which probably enhances non-shivering thermogenesis [88]. It is known that continuous cold stress increases generation of pro-oxidants in thermogenic tissues. Despite the increased activity of antioxidant enzymes, liver from cold-exposed rats displayed decreased total anti-oxidant capacity and increased oxidative damage to lipids and proteins [89]. Possibly, that is why the list of genes expressed in the liver was enriched with the terms "Regulation of reactive oxygen species metabolic process" and “Transition metal ion transport” (*SLC30A10, ABCC2, STEAP3 XDH,* and *AGXT2* genes). The class “Bile acid secretion” (*ABCB4* and *ABCC2* genes) was likely related to the nature of nutrition of mammals in Arctic and Antarctic regions, particularly to fat-rich diets.

## Conclusion

Here we present a compendium comprising of 416 mammalian genes involved in adaptations to cold environments. These 416 genes were revealed based on the intersections of positively selected gene lists from six mammalian species dwelling in Arctic and Antarctic environments. Only genes found independently selected in at least two species were included into the compendium. Our analysis did not reveal genes involved in cold adaptation that would be shared by all six species. However, our study points to the existence of general mechanisms and biochemical pathways of cold adaptation (reorganization of the cardiovascular system, increased thickness and strength of the skin, increased heat production, increased immunity, behavioral changes). It should be noted that in addition to published data on other mechanisms of cold adaptation and controlling genes, our analysis revealed that genes expressed in the skin may also play a significant role in adaptation to the cold.

## Additional file

Additional file 1: Table S1. Combined list of human genes hypothesized to be involved in cold adaptation according to Cardona et al. (2014) and Fumagalli et al. (2015). **Table S2**. Candidate genes identified to be under positive selection in Arctic fox (Kumar et al., 2015). **Table S3.** Genes potentially underwent positive selection in Yakut horse (Librado et al., 2015). **Table S4**. List of genes with fixed, derived amino acid and loss-of-function substitutions in Woolly Mammoths compared to Asian elephants (Lynch et al., 2015). **Table S5**. Candidate genes under positive selection according to Hudson-Aguade-Kreitman test in in Polar bear (Liu et al., 2014). **Table S6**. Minke whale genes found as positively selected (Yim et al., 2014). **Table S7**. List of 416 genes that potentially underwent selection in at least two different species (*Selection_shared* list, compendium). **Table S8**. Over-represented GO terms that characterize 416 genes from the compendium. **Table S9**. Gene expression analysis performed by TSEA tool. **Table S10**. Over-represented GO terms that characterize 42 genes from the compendium, which are expressed mainly in the lung; **Table S11**. Over-represented GO terms that characterize 21 genes from the compendium, which are expressed mainly in the liver. (XLSX 1315 kb)

## Abbreviations

FDR: False discovery rate
GO: Gene ontology
SNP: Single nucleotide polymorphism
